# Bayesian Geostatistical Analysis and Prediction of Rhodesian Human African Trypanosomiasis

**DOI:** 10.1371/journal.pntd.0000914

**Published:** 2010-12-21

**Authors:** Nicola A. Wardrop, Peter M. Atkinson, Peter W. Gething, Eric M. Fèvre, Kim Picozzi, Abbas S. L. Kakembo, Susan C. Welburn

**Affiliations:** 1 Centre for Infectious Diseases, Division of Pathway Medicine, College of Medicine and Veterinary Medicine, University of Edinburgh, Edinburgh, United Kingdom; 2 School of Geography, University of Southampton, Southampton, United Kingdom; 3 Spatial Ecology and Epidemiology Group, Department of Zoology, University of Oxford, Oxford, United Kingdom; 4 Centre for Infectious Diseases, Institute of Immunology and Infection Research, University of Edinburgh, Edinburgh, United Kingdom; 5 Ministry of Health, Department of National Disease Control, Uganda; Yale University, United States of America

## Abstract

**Background:**

The persistent spread of Rhodesian human African trypanosomiasis (HAT) in Uganda in recent years has increased concerns of a potential overlap with the Gambian form of the disease. Recent research has aimed to increase the evidence base for targeting control measures by focusing on the environmental and climatic factors that control the spatial distribution of the disease.

**Objectives:**

One recent study used simple logistic regression methods to explore the relationship between prevalence of Rhodesian HAT and several social, environmental and climatic variables in two of the most recently affected districts of Uganda, and suggested the disease had spread into the study area due to the movement of infected, untreated livestock. Here we extend this study to account for spatial autocorrelation, incorporate uncertainty in input data and model parameters and undertake predictive mapping for risk of high HAT prevalence in future.

**Materials and Methods:**

Using a spatial analysis in which a generalised linear geostatistical model is used in a Bayesian framework to account explicitly for spatial autocorrelation and incorporate uncertainty in input data and model parameters we are able to demonstrate a more rigorous analytical approach, potentially resulting in more accurate parameter and significance estimates and increased predictive accuracy, thereby allowing an assessment of the validity of the livestock movement hypothesis given more robust parameter estimation and appropriate assessment of covariate effects.

**Results:**

Analysis strongly supports the theory that Rhodesian HAT was imported to the study area via the movement of untreated, infected livestock from endemic areas. The confounding effect of health care accessibility on the spatial distribution of Rhodesian HAT and the linkages between the disease's distribution and minimum land surface temperature have also been confirmed via the application of these methods.

**Conclusions:**

Predictive mapping indicates an increased risk of high HAT prevalence in the future in areas surrounding livestock markets, demonstrating the importance of livestock trading for continuing disease spread. Adherence to government policy to treat livestock at the point of sale is essential to prevent the spread of sleeping sickness in Uganda.

## Author Summary

The tsetse transmitted parasites, *Trypanosoma brucei rhodesiense* and *Trypanosoma brucei gambiense*, cause the fatal disease human African trypanosomiasis (HAT); the clinical progression, as well as the preferred diagnostic and treatment methods differ between the two types. Currently, the two do not overlap, although recent spread of Rhodesian HAT in Uganda has raised concerns over a potential future overlap. A recent study using geo-referenced HAT case records suggested that the most recent spread of Rhodesian HAT may have been due to movements of infected, untreated livestock (the main reservoir of the parasite). Here, the initial analysis has been extended by explicitly accounting for spatial locations and their proximity to one another, providing improved accuracy. The results provide strengthened evidence of the significance of livestock movements for the continued spread of Rhodesian HAT within Uganda, despite the introduction of cattle treatment regulations which were implemented in an effort to curb the disease's spread. The application of predictive mapping indicates an increased risk of HAT in areas surrounding livestock markets, demonstrating the importance of livestock trading for continuing disease spread. This robust evidence can be used for the targeting of disease control efforts within Uganda to prevent further spread of Rhodesian HAT.

## Introduction

The geographical ranges of Rhodesian human African trypanosomiasis (HAT, also known as sleeping sickness), caused by the *Trypanosoma brucei rhodesiense* parasite, and the Gambian form of the disease, caused by *Trypanosoma brucei gambiense* are not believed to overlap, and Uganda is the only country thought to support transmission of both diseases within its borders [1]. Since the 1980s, Rhodesian HAT has spread into eight districts in Uganda which have not previously supported transmission [1]–[6], narrowing substantially the zone currently distancing it from endemic foci of Gambian HAT [1]. Both forms of HAT are transmitted by tsetse flies (*Glossina* spp), and are fatal if untreated, although the speed of progression to death varies between the two (within approximately six months for Rhodesian HAT compared with years for Gambian HAT). A reservoir of infection is present for *T. b. rhodesiense* (predominantly livestock in Uganda) in contrast with *T. b. gambiense* for which no known reservoir exists other than humans. As a result, the most effective control options for the two forms of the disease differ, as do diagnostic procedures and treatment regimes. Currently, treatment is implemented based on knowledge of the areas affected by each type of HAT; Gambian HAT occurs in the north west of Uganda and Rhodesian HAT in the south east. Medical staff in endemic areas will presume infection is caused by the subtype known to exist in that area and implement the appropriate treatment regimen. A definitive diagnostic differentiation between the two parasite subtypes is difficult and requires expensive, complex methods which are not currently available in affected areas. Consequently, spatial concurrence of the two forms of HAT would compromise diagnostic and treatment protocols, resulting in a higher proportion of treatment failures and placing increased pressure on an already stretched health system [7].

Recent research has focused attention on the environmental and climatic variables involved in the spatial distribution and spread of Rhodesian HAT [4], [6], [8], [9]. It is believed that the spread of Rhodesian HAT into Tororo district in 1984 was encouraged by the political and economic situation in the country during an epidemic which began in the early 1970s. A lack of resources and trained personnel, insufficient control efforts and large volumes of uncontrolled population movements through tsetse infested bush ultimately led to the spread into Tororo district [2], [3], [10]. Subsequent movement further north west in 1998, into Soroti district, was attributed to the trading of untreated, infected livestock from *T. b. rhodesiense* endemic areas at a local livestock market [4]. Since this finding, regulations have been introduced requiring the treatment (with trypanocidal drugs) of all cattle from endemic areas prior to the issue of transport permits [11]. However, the further subsequent spread into Kaberamaido and Dokolo districts in 2004 has raised questions over the implementation of these regulations, with a recent study indicating that this new expansion could also be due to the movement of untreated livestock from endemic areas [6].

It is well documented that the focal distribution of human HAT is determined largely by the ecological and environmental requirements of the tsetse fly vectors [8], [12]–[14]. There is a wide range of *Glossina* species present across the sub-Saharan fly belt; within the areas of Uganda affected by Rhodesian HAT, the predominant species of tsetse vector is *Glossina fuscipes fuscipes*, which is restricted to riverine vegetation habitats (patches of vegetation on the banks of rivers, lakes or wetlands) [15], [16]. Several studies have used a combination of ground measured and satellite-derived variables to investigate and quantify relationships between the occurrence of tsetse fly vectors and external factors (i.e. environmental or climatic). Environmental factors demonstrated to have a significant influence on vector density include vegetation cover, land use patterns, land cover types, normalised difference vegetation index (NDVI; a surrogate measure for the greenness of vegetation) and rainfall [12], [14], [17]–[21]. However, the observed relationships vary between vector species, studies, and geographical areas, highlighting the importance of local factors in vector occurrence.

The dependence of HAT transmission on the availability of competent vector populations leads to indirect associations between the spatial distribution of HAT and a variety of environmental and climatic factors. Within affected regions, areas with high HAT incidence tend to occur where there is a lot of contact between humans, tsetse flies and animal reservoirs (for Rhodesian HAT), for example, watering points [17]. Increased disease risk and the spatial clustering of cases have been observed in areas close to wetlands and swamps, highlighting the importance of tsetse habitat requirements within the study areas [8], [13]. A comprehensive understanding of the social, environmental and climatic aspects involved in the spatial distribution and spread of Rhodesian HAT is vital to enable the targeting of disease control activities. The interaction of factors influencing the long distance spread of Rhodesian HAT (e.g. livestock trading networks) with the environmental and climatic factors controlling the spatial distribution of the disease in affected areas is not well understand. Such knowledge could provide significant evidence for targeted control measures to prevent the further spread and establishment of the disease.

Batchelor *et al* [6] conducted a preliminary study of the spatial distribution of Rhodesian HAT in two of the most recently affected districts of Uganda (Kaberamaido and Dokolo) using two variations of a logistic regression method. A one-step logistic regression modelling the prevalence of HAT was compared with a two-step method which modelled the occurrence of HAT, delineated areas with a high predicted probability of occurrence, and then modelled the prevalence of HAT within these areas only. Both methods detected significant correlations between the occurrence or prevalence of HAT and external variables including distance to the closest livestock market, distance to the closest health centre, maximum normalised difference vegetation index (NDVI a measure of the amount of green vegetation) and minimum land surface temperature (LST) as well as several other environmental variables. The results indicated that the spread of HAT into this area may have been facilitated by the continuing trade of untreated, infected livestock from endemic areas as has been previously demonstrated in neighbouring Soroti district [4]. However, the large number of significant variables in each model hinders the biological interpretation of covariate effects. In addition, both models were shown to over-predict prevalence of HAT, particularly in villages with an observed prevalence of zero.

The use of non-spatial methods for the analysis of data with a spatial structure can lead to biased regression parameters, underestimated standard errors, falsely narrow confidence intervals and, thus, an overestimation of the significance of covariates [22]–[24]. Residual analysis following the logistic regression modelling indicated the presence of some residual spatial autocorrelation (where observations separated by small distances are more alike than observations separated by larger distances) after accounting for covariate effects, signifying that the variables used in the models did not capture all of the spatial variation in the observations [6]. The current research employs a spatial extension of the one-step logistic regression analysis. Specifically, a generalised linear geostatistical model in a Bayesian framework was used to account explicitly for spatial autocorrelation and incorporate uncertainty in the input data and model parameters. The current analysis provides a more rigorous analytical approach, potentially resulting in more accurate parameter and significance estimates and increased predictive accuracy [22], [25], [26]. The validity of the hypothesis that the movement of untreated, infected livestock resulted in the introduction of *T. b. rhodesiense* to the study area was assessed, given a more robust parameter estimation and appropriate assessment of covariate effects. The application of such methods to epidemiological research for the estimation of covariate effects and predictive mapping has been demonstrated in several recent studies including Diggle *et al* [27], Hay *et al* [28], Craig *et al* [29] and Kazembe *et al* [30].

## Materials and Methods

### Ethics Statement

No patient names were recorded to maintain patient confidentiality and to adhere to the International Ethical Guidelines for Biomedical Research Involving Human Subjects. The use of these data was approved by the University of Edinburgh Research Ethics Committee.

### Study area

The area of study included Kaberamaido and Dokolo districts (in the Eastern and Northern regions respectively) in Uganda, which have been affected by Rhodesian HAT since 2004. The study districts border the northern shore of Lake Kyoga with a combined area of approximately 2,740 km^2^ and a population of approximately 261,000 [31]. The main economic activities within the study area are agriculture and fishing, with the majority of the population engaged in subsistence farming [32]. The study period included Rhodesian HAT cases occurring from February 2004 (when the first cases were reported) to December 2006. A control programme involving the mass treatment of cattle in the study area began in September 2006. This control programme aimed to decrease the prevalence of human infective *T. b. rhodesiense* in the reservoir and, thus, altered the epidemiology of HAT in this area in the subsequent year; hence we have excluded from the analysis any cases diagnosed after 2006.

### Human African trypanosomiasis data

Records of all patients resident within Kaberamaido and Dokolo districts that received a positive diagnosis (direct detection of the parasite in blood, lymphatic fluid or cerebrospinal fluid using microscopy) of HAT between January 2004 and December 2006 were obtained from Lwala hospital (Kaberamaido district) and Serere hospital (Soroti district, which neighbours Kaberamaido). All villages within Kaberamaido and Dokolo districts were geo-referenced using a handheld global positioning system (GPS: Garmin, E-trex Venture), with direction from local guides. The HAT records were linked to the geo-referenced village dataset using village of residence and visualised using ArcMap 9.1 (ESRI, Redlands, CA). To maintain the anonymity of subjects and patient confidentiality and to adhere to the International Ethical Guidelines for Biomedical Research Involving Human Subjects, no patient names were recorded within the database or as part of the data collection process. Further details regarding the provenance of these data have previously been published by Batchelor *et al* [6].

### Covariate data

Non-spatial logistic regression methods were used to identify a set of environmental, climatic and social variables that were significantly correlated with HAT prevalence [6]. These were: distance to closest livestock market; distance to closest health centre; distance to closest area of woodland; maximum normalized difference vegetation index (NDVI); NDVI phase of annual cycle (the timing of the cycle); minimum land surface temperature (LST); mean LST; LST phase of annual cycle and LST annual amplitude (the amount of variation around the mean). The value of each covariate was extracted for each village in the study area.

The locations of all livestock markets and health centres within the study area were recorded during fieldwork using a handheld GPS. Maps detailing areas of woodland within the study area were obtained from the National Biomass Survey, which was conducted by the Uganda Forest Department between 1995 and 2002 [33]. These classifications were the result of a quantitative interpretation of remotely sensed images along with ground data and supplementary data layers and, thus, their accuracy may be variable. The distance between each village and the closest livestock market, health centre and area of woodland was calculated in kilometres. The LST and NDVI indices were derived using a Fourier transformation of Advanced Very High Resolution Radiometer (AVHRR) imagery. NDVI is a measure of the amount of green vegetation [34]; both vegetation cover (in terms of suitable tsetse habitat) and temperature have been shown to influence the distribution of HAT [12]. Temporal Fourier processing reduces the number of data to be processed by eliminating redundancy and characterising seasonality. Full details regarding the data used and the Fourier analysis can be found in Hay *et al* [35].

### Statistical analysis

The spatial variation in HAT prevalence within Kaberamaido and Dokolo districts was modelled using model-based geostatistics [26], [36] with a spatial generalised linear model and Bayesian inference of model parameters [36]. The method used was a spatial extension of a logistic regression model, which can be used for the analysis of geo-referenced binomial data (e.g. disease prevalence where the outcome variable is bounded between zero and one) [26], [36], [37]. The modelling process describes the variability in the response variable as a function of the explanatory variables with the addition of a stochastic spatial effect to model the residual spatial autocorrelation [26], [37]. Exponentiation of the model parameters gives the odds ratio (OR) for each covariate; this indicates the strength and direction of relationships between the explanatory and outcome variables.

### Model specification

The total number of HAT cases 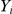 within village 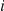 was modelled as a conditionally independent binomial variable, 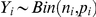, where 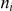 is the total village population and 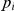 is the underlying population prevalence of HAT at location 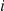. The method is an extension of a GLM using the logit link function, incorporating a stochastic spatial effect *S*(**x**) as follows:
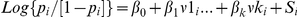
where 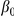 is the intercept term and 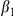 to 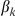 are the regression coefficients relating to covariates 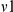 to 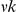.

The stochastic spatial component is modelled as a zero mean Gaussian process with variance 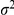 and autocorrelation function 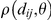, where 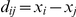 measures the Euclidean distance between 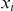 and 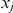 ; 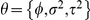; 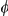 is the range (effectively the maximum distance at which there is spatial autocorrelation between observations) and 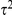 is the relative nugget. This serves to model the spatial variation in the residuals after accounting for the covariates, 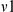 to 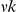.

The model parameters were estimated using a Bayesian framework with a Markov chain Monte Carlo (MCMC) algorithm in the package *geoRglm* [37] in the R statistical software [38]. MCMC methods involve the construction of a Markov chain (a mathematical representation of a random process where future values are conditionally independent of past values, and depend only on the present value), with the desired probability distribution at its equilibrium (the stationary posterior distribution which the chain will converge to following a suitable number of iterations). Samples can then be drawn from the equilibrium distribution and summarised to provide parameter estimates, quantiles and other measures of the distribution [39].

Priors (in Bayesian inference a prior is a probability distribution expressing uncertainty about a parameter before taking into account data observations) were selected for each parameter to represent prior knowledge of their distributions. Non-informative, uniform priors were selected for the regression parameters, 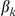 and the variance, 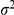 in the absence of prior knowledge. This allows the observed data to have the greatest influence on posterior distributions without being constrained by the choice of prior and can also improve MCMC convergence [27], [40]. The range parameter, 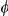, was fixed and optimised by minimising the mean squared error due to potential problems with the estimation of both 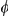 and 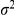 [41]. This acted as a compromise between statistical rigor and computational ease: the range parameter would ideally be estimated along with all other parameters, but previous trials indicated problems with the mixing and convergence of MCMC chains when both 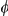 and 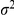 were being estimated. A Matérn correlation function was used [27] with a discrete order (smoothness parameter) of 0.5. This equates to the use of an exponential correlation function. The relative nugget parameter, 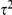, was fixed at 1 after inspection of the residual variogram.

### Univariate parameter estimation

Due to convergence and mixing problems when including all of the covariates listed in Table 1, each of the explanatory variables was examined independently using the above modelling framework. The MCMC algorithms were tuned to give an acceptance rate of approximately 60%, and the fixed prior for 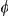 was optimised using several iterations to obtain a minimised mean squared error for each explanatory variable.

**Table 1:** Odds ratios and 95% CrI from Bayesian univariate regression analysis.

	Odds Ratio	95% CrI
**Distance to closest livestock market**	0.81	0.76 to 0.86*
**Distance to closest health centre**	0.85	0.76 to 0.95*
**Maximum NDVI**	1.06E^−5^	2.75E^−12^ to 36.60
**Minimum LST**	1.57	1.11 to 2.29*
**LST phase of annual cycle**	1.19	0.91 to 1.51
**Distance to woodland**	1.19	0.95 to 1.46
**NDVI phase of annual cycle**	3.25	1.02 to 10.07*
**Mean LST**	0.90	0.59 to 1.38
**LST annual amplitude**	0.86	0.66 to 1.13

*Indicates significance at the 95% level.

The univariate spatial models were run for 2,000,000 iterations, with the first 1,000,000 discarded and every 100^th^ iteration thereafter stored to assess the significance of each explanatory variable. Convergence and mixing of the MCMC algorithms was judged based on traceplots and autocorrelation plots for each model parameter to ensure that convergence had been reached, the chains had mixed adequately and autocorrelation amongst the samples was minimal. The mean values from the posterior distribution and their 95% credible intervals (CI)s were calculated and exponentiated to provide odds ratios (OR)s and their respective uncertainty measures. Only those covariates that were significantly associated with HAT prevalence (i.e. the 95% CI for the OR did not include the value 1) were selected for the multivariate spatial regression model.

### Multivariate parameter estimation

An initial run of the multivariate model was carried out following the optimisation of 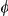 and tuning of the MCMC algorithm as described for the univariate parameter estimation. The regression parameters and 95% CIs were inspected. Any covariates that were non-significant in the multivariate model were discarded from the final model.

The fixed 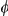 value was again optimised for the final multivariate model and the MCMC algorithm tuned. Following a burn-in of 1,000,000 iterations, the chain was run for a further 5,000,000 iterations, with every 1000^th^ iteration thereafter stored, resulting in a total of 5,000 samples from the posterior distributions. The regression parameters and 95% CIs were obtained from the model and exponentiated as above.

### Spatial predictions

A 2 km spatial resolution prediction grid was created for the study area, containing covariate values at each prediction location (grid cell). Samples from the predictive distribution for each prediction location were generated using the MCMC algorithm given the explanatory variables at each grid cell. The posterior medians and lower and upper 95% CI limits from the predictive distributions were extracted to give predicted prevalence and uncertainty estimates at all locations. The predictions were then exported to ArcMap for illustrative purposes.

A scatter plot of predicted prevalence versus observed prevalence was created to illustrate the relationship between the model predictions and observations, and the correlation between fitted and observed prevalence was calculated. In addition, the mean error, median error and absolute mean error (calculated using prevalence per 100 population and, therefore, expressed as percentages) were calculated based on the difference between observed and predicted prevalence at each location, to give an indication of the prediction bias (mean and median error) and accuracy (absolute mean error). The Pearson residuals were calculated [42] and the residual variogram was plotted to examine any residual spatial autocorrelation. This was compared with the residual variogram from the non-spatial logistic regression model as described in Batchelor *et al* [6].

## Results

There were a total of 692 villages within the study area (Kaberamaido and Dokolo districts); all but two were geo-referenced (two were excluded due to logistical difficulties). Of the remaining 690 villages, 18 that had recently separated into two were merged for the purpose of the analysis. Within the study period 354 cases of HAT were reported from these two districts, which equates to an overall period prevalence (2004–2006) of 0.14 per 100 population, although this value is very likely to be an underestimate due to complex issues surrounding care seeking behaviour for HAT and the under utilisation of health services [43], [44]. Of these patient records, 52 could not be matched to any of the known villages in the study area and so were excluded from the analysis. This was most likely due to inaccuracies in the recording of patient details in the hospital records. Treatment outcomes were not recorded for all cases; of the 251 cases for which the treatment outcome was known, 93.6% were treated successfully and 6.4% died.

### Univariate parameter estimation

From the univariate spatial regression model, five variables which were significantly correlated with HAT prevalence using deterministic, non-spatial logistic regression did not retain their statistical significance (see Table 1 for ORs and 95% CIs). Four covariates retained their significance in the spatial regression analysis. Increasing distance from the closest livestock market had a protective effect in terms of HAT prevalence (OR = 0.81, 95% CrI = 0.76 to 0.86), as did increasing distance to the closest health centre (OR = 0.85, 95% CrI = 0.76 to 0.95). Areas with higher minimum land surface temperature and larger NDVI phase of annual cycle had significantly increased odds of HAT (OR = 1.57, 95% CrI = 1.11 to 2.29 and OR = 3.25, 95% CrI = 1.02 to 10.07 respectively).

### Multivariate parameter estimation

The NDVI phase of annual cycle covariate did not retain statistical significance when included along with the other three significant covariates in the multivariate spatial regression (OR = 1.73, 95% CrI = 0.61 to 5.00), and so was omitted from the final multivariate model. The remaining three covariates retained significance at the 95% level (see Table 2). Both increasing distance to the closest livestock market and increasing distance to the closest health centre had protective effects (OR = 0.83, 95% CrI = 0.78 to 0.88 and OR = 0.88, 95% CrI = 0.79 to 0.97 respectively). Additionally, areas with a higher minimum LST had increased odds of HAT (OR = 1.49, 95% CrI = 1.09 to 2.10). The variance (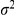) was estimated to be 1.17 (95% CrI = 0.74 to 1.75).

**Table 2:** Odds ratios and 95% credible intervals from Bayesian multivariate regression analysis.

	Odds ratio	95% CrI
**Intercept**	9.02E^−6^	4.77E^−8^ to 0.001
**Distance to closest livestock market**	0.83	0.78 to 0.88
**Distance to closest health centre**	0.88	0.79 to 0.97
**Minimum LST**	1.49	1.09 to 2.10
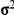 **(variance)**	1.17*	0.74 to 1.75

*Indicates variance value rather than OR.

The posterior distributions for all parameters were normally distributed, although for *σ^2^* there was a slight positive skew. Traceplots and autocorrelation plots for model parameters were examined to assess the mixing and convergence of the MCMC algorithms and each appeared to have reached convergence during the burn-in period and to be mixing well. Autocorrelation amongst samples was minimal. The posterior distribution curves for the final model parameters (Figure S1), traceplots (Figure S2) and autocorrelation plots (Figure S3) are available in the supplementary information.

### Spatial predictions

**Figure 1:**
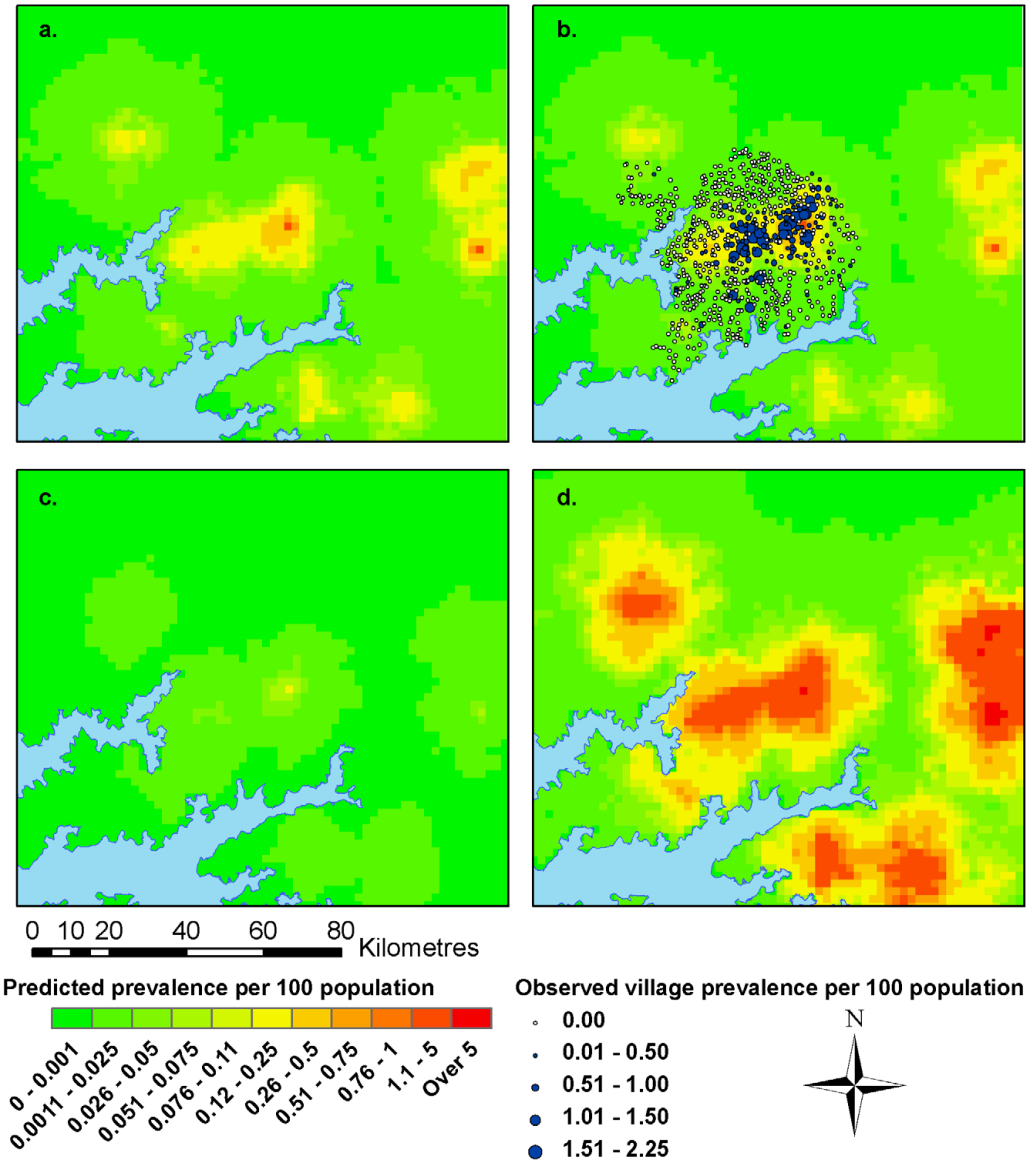
Predicted prevalence of HAT per 100 population from final spatial model. Predicted prevalence (1a), predicted prevalence with observed village prevalence (1b) and lower (1c) and upper (1d) 95% credible limits.

The predicted prevalence surface from the final spatial model is illustrated in Figure 1a and is overlaid with observed village prevalence data in Figure 1b (displayed as prevalence per 100 population; a percentage). Figures 1c and 1d illustrate the lower and upper 95% credible limits for the prediction. The area of highest predicted prevalence within the study area corresponds with the majority of high prevalence villages. Several potential high prevalence areas out with the study area correspond to areas surrounding livestock markets, with the effect of distance to the closest health centre and minimum land surface temperature also accounted for. The areas with the highest predicted prevalence also have the largest 95% credible intervals, which is due to the greater variability of observed village level prevalence in the high prevalence areas (i.e. villages with high prevalence are interspersed with zero prevalence villages within the high prevalence area).

**Figure 2:**
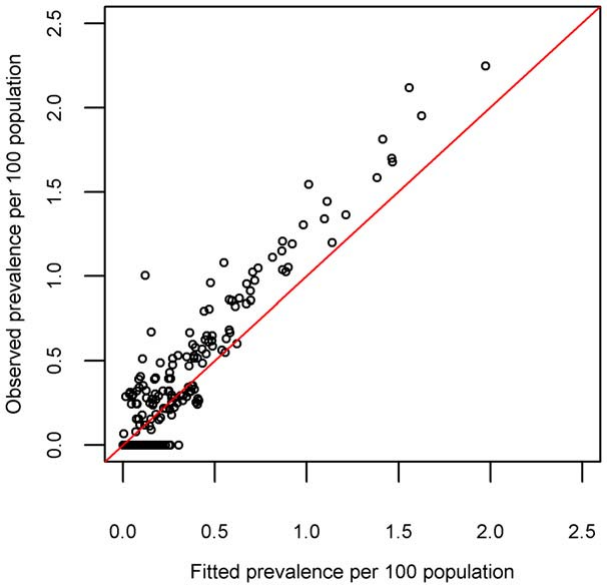
Fitted village prevalence versus observed village prevalence.

A plot of predicted prevalence versus observed prevalence (Figure 2) shows a tendency to under-predict the prevalence in high prevalence villages and over-predict in zero prevalence villages, with an overall correlation between observed and fitted prevalence of 0.95. The predicted prevalence (expressed as prevalence per 100 population; a percentage) had a mean error of −0.00094%, a median error of 0.018% and an absolute mean error of 0.064%.

**Figure 3:**
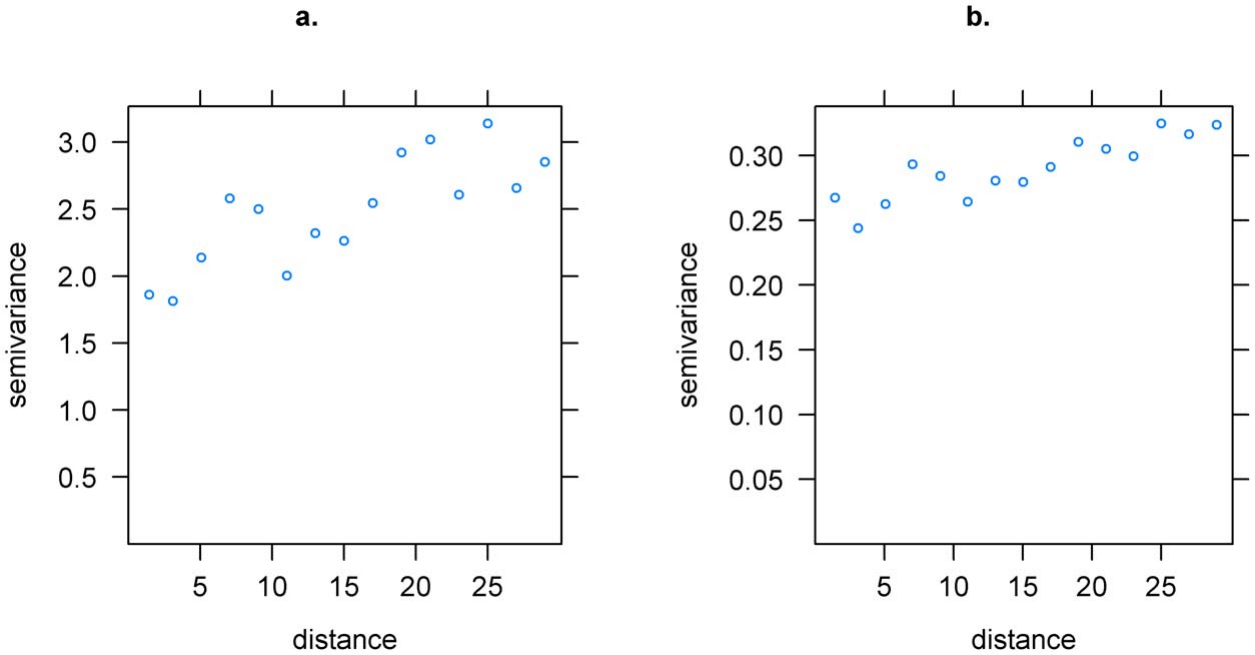
Residual variograms using Pearson residuals. Residual variogram from the non-spatial model (3a) [6] and the Bayesian spatial model (3b).

The empirical variogram of the Pearson's residuals from the non-spatial model as discussed in Batchelor *et al* [6] indicates the presence of some unexplained spatial variation in the residuals (Figure 3a). The residual spatial autocorrelation from the spatial model (Figure 3b) gives a flatter variogram, with a smaller amount of residual variation than the non-spatial model indicating that the spatial model has accounted for a larger amount of the spatially correlated variation in the prevalence data than the non-spatial model. Overall, the diagnostics show that although the spatial model results in less residual variation and greater correlation between observed and predicted prevalence, there is still some residual spatial variation in HAT prevalence within the study area which is not being accounted for; in particular, several zero-prevalence villages have higher predicted prevalence than was observed.

## Discussion

The results presented here extend an initial (non-spatial) analysis as published by Batchelor *et al* [6]. A spatial analysis was conducted, in which a generalised linear geostatistical model was applied with Bayesian implementation, as described by Diggle *et al* [26]. This method allows the assessment of covariate effects while modelling the residual spatial autocorrelation explicitly and incorporating uncertainty in the input data and model parameters. This approach allowed a more robust assessment of covariate effects, with more accurate parameter and significance estimates than those obtained using non-spatial methods. The results provide significant support for the hypothesis that Rhodesian HAT was introduced into Kaberamaido and Dokolo districts via the movement of infected livestock. In addition, the significant relationships between HAT prevalence and environmental, climatic and social factors detected using the non-spatial regression have been clarified.

Following on from the non-spatial logistic regression methods discussed in Batchelor *et al* [6], many of the covariates that retained significance in the multivariate non-spatial regression model of HAT prevalence lost significance in the Bayesian implementation of a spatial logistic regression model. As a starting point for the spatial model, it would have been preferable to include all covariates from the final fitted non-spatial logistic regression model. Any covariates which did not retain statistical significance when accounting for residual spatial autocorrelation would then be removed prior to the final fitting of the model. However, when including all covariates, problems with the convergence and mixing of the MCMC algorithms were encountered. It is likely that the problematic MCMC performance with the full multivariate model was due to correlation and redundancy of some of the covariates and potentially also difficulties in estimating a large number of parameters at the same time.

Five covariates did not retain statistical significance during the univariate spatial regression and one (NDVI phase of annual cycle) did not retain significance during the multivariate spatial regression, indicating that the non-spatial model may have inflated the significance of covariates and produced inaccurate parameter estimates. The final spatial model included three covariate effects: distance to the closest livestock market, distance to the closest health centre and minimum LST. These results, using a more robust assessment of covariate effects, provide considerable strength to the hypothesis that the movement of infected, untreated livestock from endemic areas resulted in the introduction of *T. b. rhodesiense* to Kaberamaido and Dokolo districts. Previous research has established that the introduction of Rhodesian HAT transmission within Soroti district (which neighbours the study area) was due to movements of untreated cattle from endemic areas through a local livestock market [4]. The results discussed here, supported by the findings discussed in Batchelor *et al* [6], strongly indicate a similar occurrence in Kaberamaido and Dokolo districts; *T. b. rhodesiense* is likely to have been introduced to Dokolo and Kaberamaido via the continued movement of untreated livestock, despite the introduction of a law requiring the treatment of livestock from endemic areas, prior to sale [11].

Within the study area, it is problematic to separate the effects of differential utilisation of the HAT treatment centre, where those living closer are more likely to travel there for diagnosis and treatment than those living further away, from the purposeful siting of the treatment centre within the area most affected by HAT. Following the detection of a number of cases in Kaberamaido district in 2004, appropriate training and equipment were provided to one hospital within the area. The facility was selected based on a number of criteria, including the location within the affected area. Due to this difficulty, the distance to the closest health centre of any kind was used rather than distance to the HAT treatment centre. The significance of this variable in the spatial regression model highlights the importance of accessibility to health services as has been shown previously [8], [43]. The observed protective effect of living further from a health centre may indicate a confounding effect, with individuals living in more remote areas and further from health care services being less likely to access treatment and, thus, be diagnosed with and treated for HAT.

Minimum LST was observed to be a risk factor for HAT, with higher prevalence in areas with higher minimum LST. Minimum LST is calculated using measurements of radiance modified by the atmosphere in several spectral wavebands and varies depending on climate and also landcover properties (e.g. amount of vegetation, urbanisation or soil moisture) [45]. The size of the study area (approximately 60 km by 60 km) suggests that the observed correlations are more likely to be due to the heterogeneous landcover profile and soil and vegetation moisture content than to climatic variability across the two districts, although the precise interpretation of this mechanism is not clear. Further work is planned to disentangle the effects of climate and landcover; utilising finer spatial resolution climatic data and encompassing a larger study area, the research will investigate the dynamic nature of the distribution of HAT and relate this to climatic, environmental and social covariates (including temperature, rainfall and landcover classes).

When the performance of the spatial regression model was compared with that of the non-spatial model (one-step model of prevalence as discussed in Batchelor *et al* [6]), the predictions from the spatial model are seen to be more accurate. The correlation between observed and fitted prevalence for the non-spatial model was 0.58, compared with a correlation of 0.95 for the spatial model. The absolute mean error for the non-spatial model was 0.13%; double that of the spatial model (0.064%, calculated based on prevalence per 100 population). Despite the increase in accuracy gained by modelling the residual spatial autocorrelation after accounting for covariate effects, there was still a tendency to over-predict in zero prevalence villages and also to under-predict in high prevalence villages. The over-prediction in zero prevalence villages indicates the presence of extra-binomial variation (greater variability in the observations than can explained by the model) whereby additional unmeasured factors may be influencing the spatial heterogeneity of HAT prevalence within small areas. From the observed prevalence it can be seen that within the main ‘focus’ of infection there are several zero prevalence villages interspersed amongst high prevalence villages, which are not explained adequately by the spatial regression model. The estimates of model uncertainty (95% CrIs) also highlight this, with larger predictive uncertainty in the areas with higher predicted prevalence as can be seen in Figures 1c and 1d. This non-constant variance in the error is known as heteroscedasticity. Future research as described above aims to deal with these issues by utilising a wider range of covariate datasets, with finer spatial resolutions.

Although these methods have taken into account the effect of health care accessibility on the spatial distribution of reported HAT, underreporting is well documented [43], [44], with evidence suggesting that for every Rhodesian HAT case that dies within the health care system, another 11 cases will go undetected and therefore untreated, resulting in death [44]. Underreporting of HAT causes serious problems for the estimation of disease burden, determination of the spatial extent of disease transmission and the prioritisation of resources, and also impacts on research conducted using data acquired from passive case detection. However, the lack of a rapid, cheap and easy to use diagnostic test for *T. b. rhodesiense*, combined with the very low prevalence of disease in affected areas, makes active screening a difficult and expensive task for the detection of very few cases of disease. Further work which is currently being planned includes active population screening in a sample of villages; this data will be compared with hospital records to ascertain the proportion of Rhodesian HAT patients that are not accessing treatment and to allow estimation of the true burden of disease in affected areas.

The research described utilised a variety of data sources providing information relevant to the distribution of the tsetse fly vector and, thus, also the distribution of Rhodesian HAT. However, accurate tsetse distribution or density data were not available for the study area, although the explicit inclusion of information on the spatial distribution of tsetse may have resulted in improved predictive power and provided further information on the determinants influencing the spatial heterogeneity in HAT prevalence within the main focus of disease. Additional factors that may play an important role in the observed spatial heterogeneity of HAT within Uganda include demographic factors, migration and human movement and behaviour patterns, due to their influence on the frequency of interaction between humans, tsetse and livestock. Although human migration has the potential to introduce *T. b. rhodesiense* to previously unaffected areas, in this situation it seems unlikely to have occurred due to the strong evidence supporting the theory of introduction via livestock movements. Additionally, the transmission of *T. b. rhodesiense* normally occurs between reservoir hosts (i.e. cattle) with only sporadic transmission to humans [46], [47].

The current research has demonstrated the application of Bayesian geostatistical modelling to the spatial distribution of HAT within a small area of Uganda. The more robust results provide strengthened evidence of the role of livestock trade in the continued spread of Rhodesian HAT within Uganda and the utility of this methodology for the prediction of HAT prevalence based on external covariates has also been demonstrated. The dataset used in this situation covered a relatively small area (two districts) with as complete a dataset as possible (all but two villages were geo-referenced, and all HAT cases that could be matched to a village of residence were used). The predictive power of this model over larger areas (i.e. out with the initial study area) is constrained due to the limited area from which the observed data came. To allow the full exploitation of these methods, future work will focus on a larger study area using a sample of villages. This will allow an investigation of HAT prevalence in relation to wider covariate ranges and will allow extrapolation over larger areas. The Bayesian implementation of model-based geostatistics as described here is computationally expensive and can be time consuming, but the application of such methods to epidemiological research is being assisted by a growing base of knowledge and expertise, along with the creation of more efficient algorithms [48], [49]. The utility of such methods for the accurate estimation of disease burden and the spatial targeting of control measures has been demonstrated in the literature by a variety of applications at local, national, regional and continental scales including malaria [50], [51], schistosomiasis [40], [52] and trachoma [53].

The research presented here illustrates the importance of spatial autocorrelation in epidemiological variables; the use of non-spatial logistic regression analysis resulted in a model with a large number of covariates, complicating the interpretation of their effects. The use of a generalised linear geostatistical modelling framework, which models the residual autocorrelation after accounting for covariate effects, gave more precise and less biased parameter and significance estimates, with only three covariates retaining significance in the final model. The Bayesian implementation of the method allowed the incorporation of uncertainty in each of the model parameters from the posterior distributions and from the definition of a random variable. By carrying out the spatial-regression analysis, the quantified relationships between HAT prevalence and significant covariates can be more confidently described and interpreted. The predictive accuracy was also increased by using the spatial regression when compared to the non-spatial logistic regression analysis. These results strengthen the evidence in support of the hypothesis generated by the analysis discussed in Batchelor *et al* [6]; that the movement of untreated, infected livestock from endemic areas resulted in the introduction of Rhodesian HAT to the study area.

## Supporting Information

Figure S1 Posterior distributions for model parameters.
(0.25 MB TIF)

Figure S2 Traceplots of MCMC output for each parameter.
(0.68 MB TIF)

Figure S3 Autocorrelation plots of MCMC output for each parameter.
(0.37 MB TIF)
