# Supplementary figures and images for: Bayesian Geostatistical Analysis and Prediction of Rhodesian Human African Trypanosomiasis
Source: PLoS Negl Trop Dis. 2010 Dec 21;4(12):e914. doi: 10.1371/journal.pntd.0000914 (PMC3006141; doi:10.1371/journal.pntd.0000914)

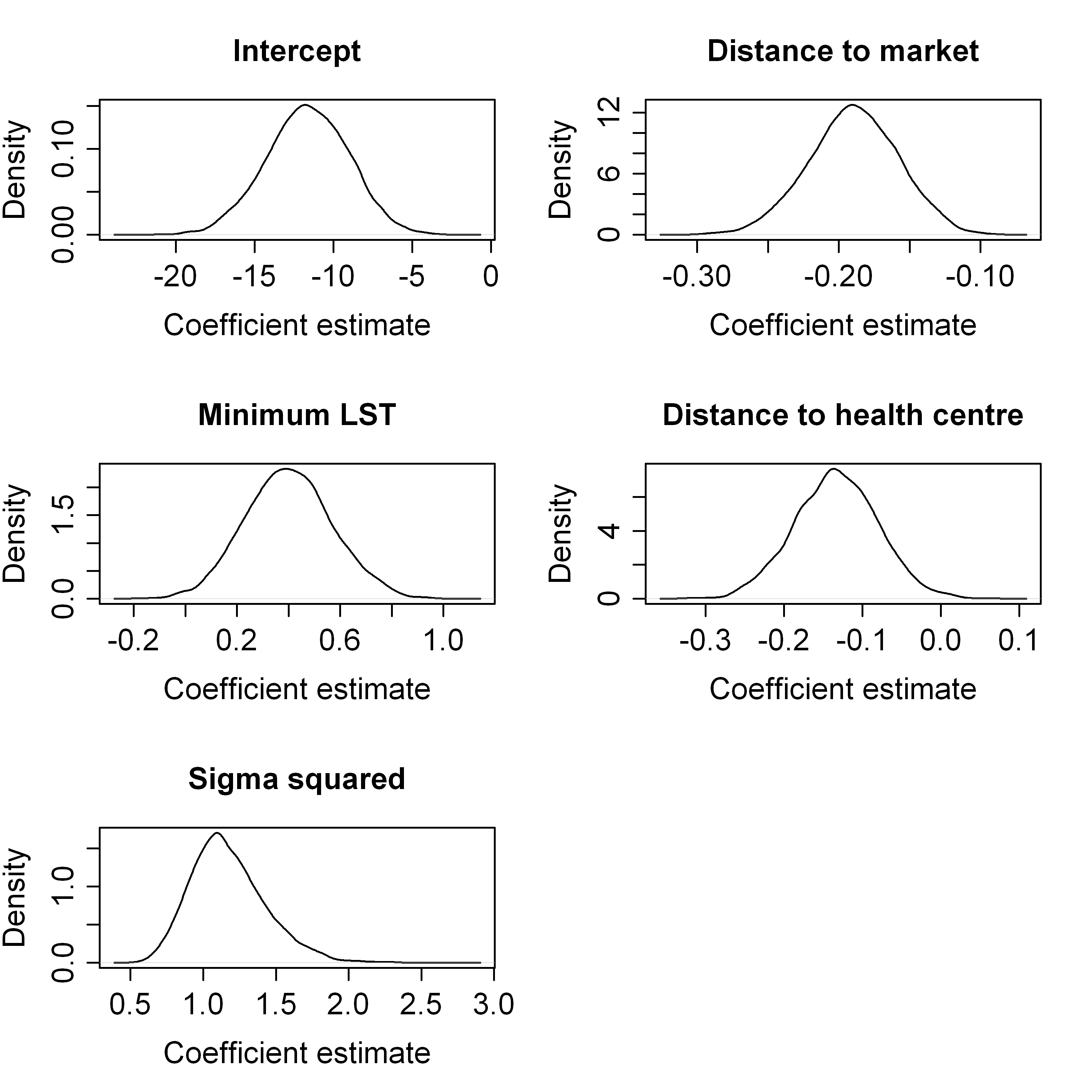

Supplement: Figure S1 — Posterior distributions for model parameters. (0.25 MB TIF) [file pntd.0000914.s001.tif]

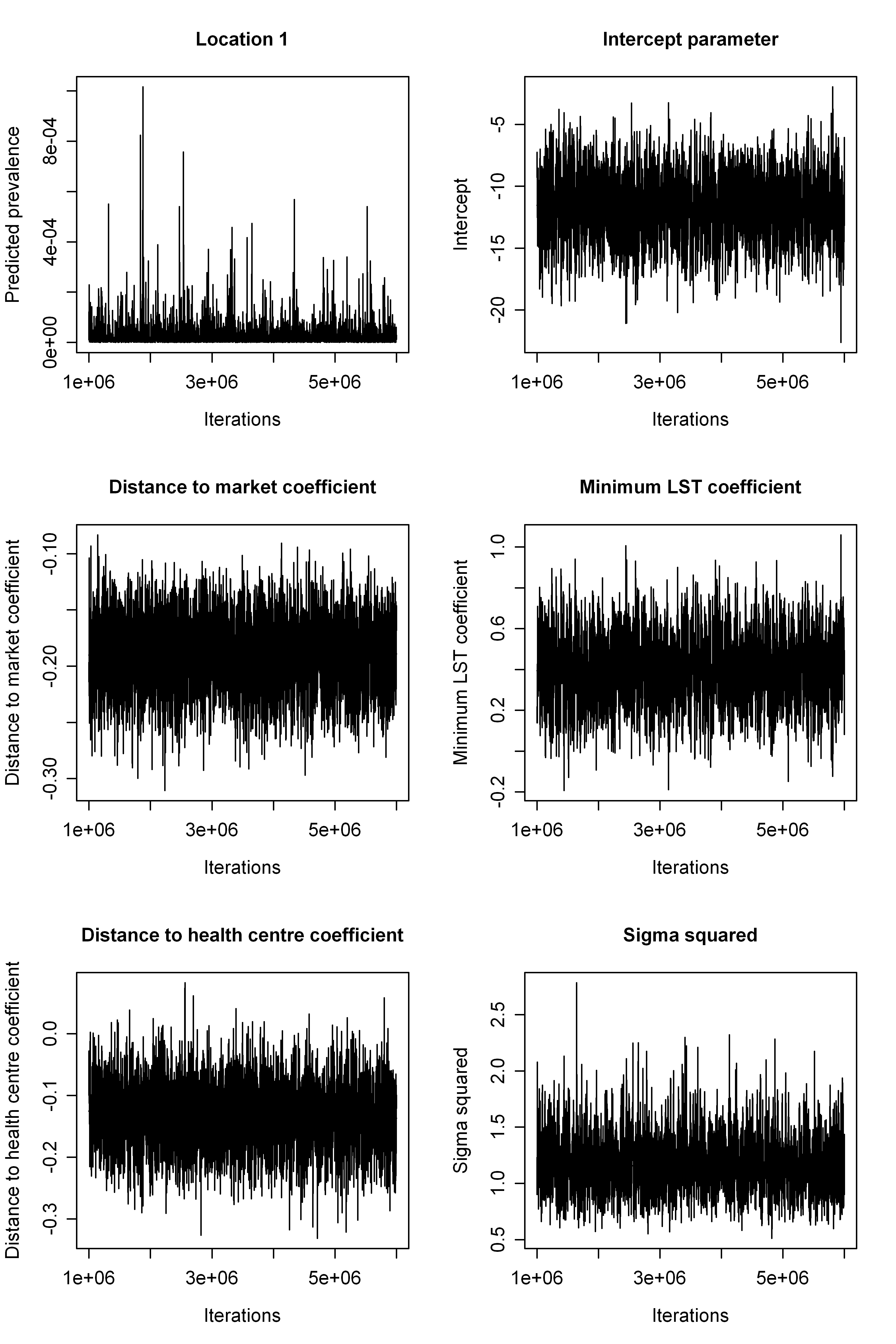

Supplement: Figure S2 — Traceplots of MCMC output for each parameter. (0.68 MB TIF) [file pntd.0000914.s002.tif]

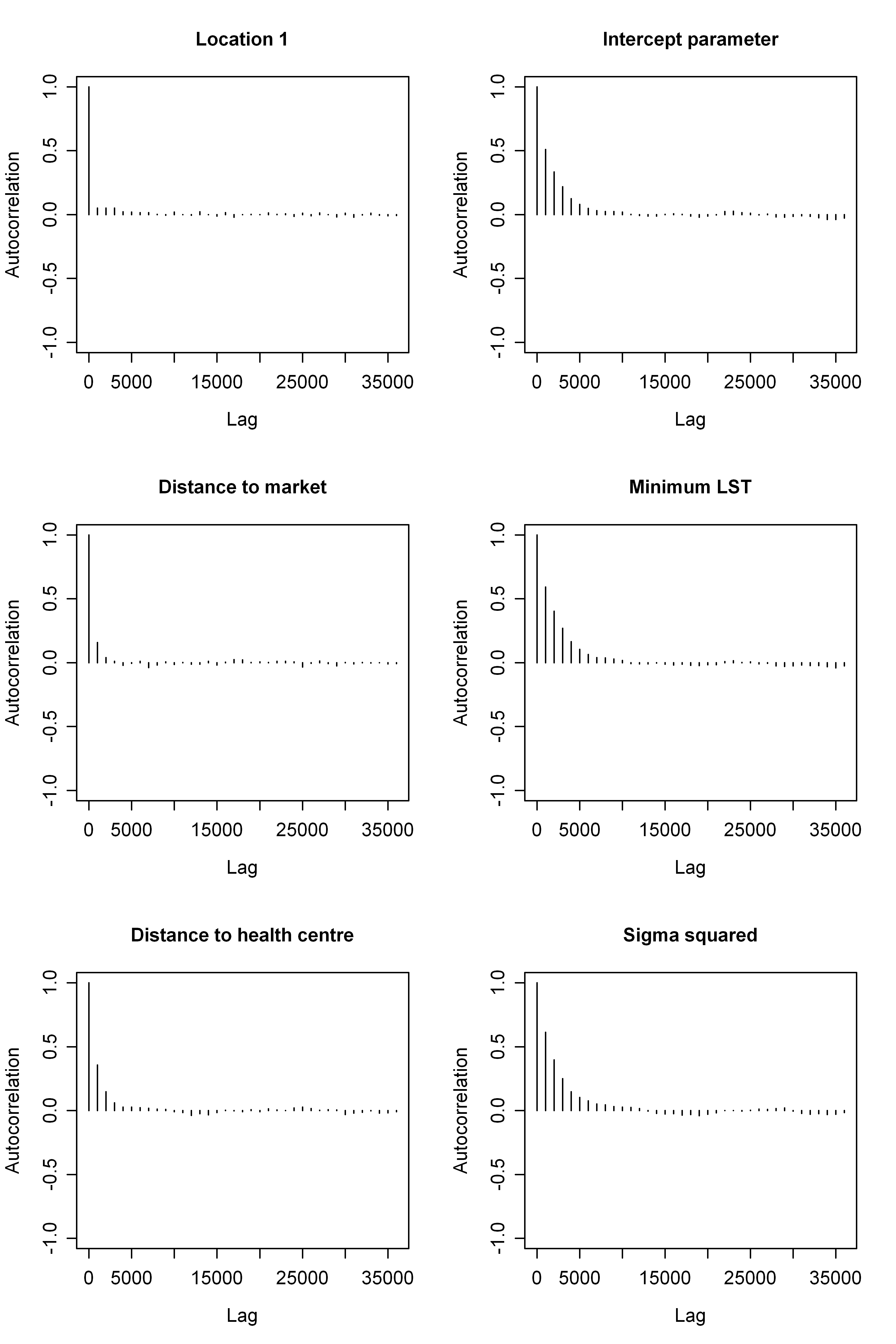

Supplement: Figure S3 — Autocorrelation plots of MCMC output for each parameter. (0.37 MB TIF) [file pntd.0000914.s003.tif]
